# A systematic review and meta-analysis of the traumatogenic phenotype hypothesis of psychosis: commentary, Alameda

**DOI:** 10.1192/bjo.2024.863

**Published:** 2025-03-03

**Authors:** Luis Alameda

**Affiliations:** Service of General Psychiatry, Treatment and Early Intervention in Psychosis Program, Lausanne University Hospital (CHUV), Lausanne, Switzerland; Department of Psychosis Studies, Institute of Psychiatry, Psychology and Neuroscience, King’s College London, National Psychosis Unit, South London and Maudsley NHS Foundation Trust, London, UK; Department of Psychiatry, Instituto de Investigación Sanitaria de Sevilla, IBiS, Hospital Universitario Virgen del Rocío, Universidad de Sevilla, Seville, Spain

**Keywords:** Childhood trauma, psychosis, traumatology, prevention, psychopathology

## Abstract

Onyeama et al have examined the clinical profile of individuals with psychosis and childhood trauma using a stringent approach that yielded selective evidence, affecting power and insight into the specific and differential roles of abuse and neglect in the clinical profile. This commentary puts the findings into a broader meta-analytical context.


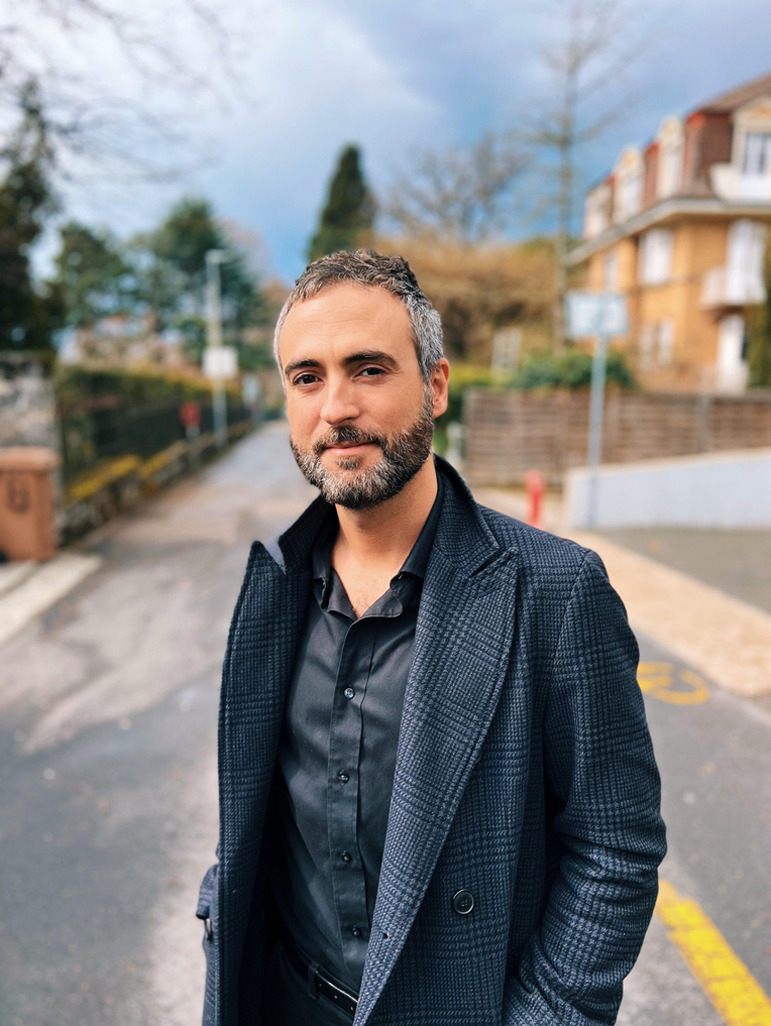


## Response

I read with interest the recent review by Onyeama et al,^1^ in which they systematically reviewed and meta-analysed quantitative and qualitative literature examining the clinical profiles of those with childhood adversity (here termed ‘developmental trauma’) and psychosis. The review is comprehensive, covering a broad range of outcomes of interest when examining the clinical profile, and it contains a very interesting section on studies examining the quality and content of delusions and perceptual abnormalities in people with psychosis and developmental trauma, adding an original and often overlooked phenomenological angle. Among the findings in this part, the authors report that people with psychosis and developmental trauma are more likely to hear voices that are thematically threatening or with content related to traumatic memories, or to have delusions that can often include persecution, although at times grandiosity or guilt as well. These findings are important and rarely reported, as not all these domains are captured by the dimensional scores of the usual scales, such as the Positive and Negative Syndrome Scale; such scales are good for scoring the severity of symptoms but poorly capture phenomenological specificities. I believe these findings are relevant for clinical practice and could have implications for psychotherapy and future research. For example, a better understanding of which factors may predispose a traumatised individual to developing delusions of guilt, as opposed to grandiosity, as a result of sexual abuse may help to guide psychological treatment in a more individualised way.

Despite these positive aspects, I believe the quantitative review uses a too conservative approach, focusing only on studies that compared participants with psychosis based on trauma levels operationalised categorically (comparing two groups of patients with and without developmental trauma, or comparing those with high versus low levels of developmental trauma, measured categorically). This limited the number of included studies (only 11 were meta-analysed overall), as the majority of the literature in the field of trauma and psychosis and associated clinical profiles uses continuous scores, such as the total score of the Childhood Trauma Questionnaire^2^ (ranging from 0 to 140), or predefined cut-offs such as those proposed by Bernstein et al, which create a score ranging from 0 to 5 (with 0 indicating no trauma and 5 indicating exposure to five different traumas^2^). These scores are suitable for capturing the dose–response relationship of types of adversity and psychosis.^3^ Moreover, a recent review of the methods used to measure adversity in psychotic disorders showed that around 30% of studies have used continuous scores.^4^ Furthermore, a meta-analysis by my group on the association between trauma and clinical profile found that only 13 of the 47 studies included formally compared two groups; the others tended to use correlations or regressions^5^ (also comparing patients with some degree of developmental trauma against those with none). I believe the stringent approach used by Onyeama et al led to a case of selective evidence affecting power and prevented the authors from examining specific aspects related to developmental trauma and clinical profiles, such as the level of specificity of the associations of abuse and neglect with clinical features. As a result, some of the findings are surprising and not in line with previous consolidated systematic evidence in the field and make the conclusions very tentative. The authors only partially discuss such findings in light of previous meta-analyses, omitting important ones on the topic^5–8^ and potentially misleading a reader not aware of what was already known as consolidated evidence on the topic of trauma and the clinical profile. The aim of this commentary was to discuss these unexpected findings, putting them into context with previous consolidated evidence in the field.

First, in terms of positive symptoms as a dimension in those with developmental trauma, the authors confirm a well-replicated finding that developmental trauma measured broadly (as total trauma including abuse and neglect experiences) is associated with more severe positive symptoms (*k* = 11), as had already been shown by Bailey et al (*k* = 18)^9^ and subsequently by my own work (*k* = 27).^5^ In their review, Onyeama et al amalgamated studies using a composite measure of developmental trauma, including any form of adversity, with those measuring subtypes such as sexual abuse (for example, Lysaker et al^10^); this will have diluted any possible level of specificity in the associations between trauma subtypes (abuse and neglect) and clinical features, preventing the authors from exploring this important aspect. Previous meta-analyses have looked at this, and there is evidence suggesting that there is are different psychopathological signatures for the effects of abuse and neglect on positive symptoms. In that regard, Bailey et al found that sexual abuse (*k* = 10) but not neglect (*k* = 5) was associated with more severe positive symptoms, a finding which I later replicated with a larger data-set (*k* = 16 for abuse and *k* = 12 for emotional neglect).^5^

A similar issue affects the findings of Onyeama et al on negative symptoms; they did not find any association with developmental trauma (measured broadly as a total trauma measure); this is not in line with previous larger meta-analyses examining the specificity of the associations between trauma subtypes and clinical domains and showing that neglect but not abuse subtypes are associated with negative symptoms.^5,9^ Third, the authors included only three studies exploring quantitatively the role of developmental trauma in delusions and found no evidence suggesting an association; this is contrary to their own qualitative findings but also those of the meta-analysis by Bailey et al, which showed an association of developmental trauma measured broadly (*k* = 10) and sexual abuse (*k* = 7) but not neglect (*k* = 5).^9^

It is important to note that it has been argued^4^ that the developmental trauma subtypes do not represent the reality of patients, as they often co-occur.^4^ It is true that if we want to explore the effects of poly-victimisation, including various forms of abuse such as sexual, physical, and emotional, as well as neglect, then putting the different forms together is acceptable – although in that case, we can only expect broad psychopathological impacts. However, some patients are only exposed to one episode of abuse (i.e. sexual) or neglect, and I believe that these are not equivalent experiences, neither involving the same mechanisms nor requiring the same trauma-informed care approach.^11,12^ Furthermore, novel data at a biological level suggest that patterns of epigenetic dysregulation (in the form of DNA methylation) associated with abuse and neglect do not overlap;^13^ levels of specificity have also been found with neuroimaging studies.^14^ Therefore, amalgamating these phenomena as equal when studying the clinical profile is, in my view, limited. Interpreting their findings, Onyeama et al make a strong case that there may be a psychotic form of post-traumatic stress disorder. I believe that if this is the case, it only applies to those with abuse experiences; for neglected patients with psychosis, a different explanation is needed.

The rest of the findings of Onyeama et al are overall in line with those of previous meta-analyses and systematic reviews in the field, showing links of developmental trauma, measured broadly, with poorer cognition,^6^ more severe dissociation,^15^ and depression and anxiety,^5^ and a lack of association with social cognition.^16^ Again, there is no mention of the differential roles of abuse and neglect in those domains.

Overall, Onyeama et al offer an interesting review of the specific phenomenological features of those with psychosis and developmental trauma. However, their quantitative synthesis uses a too stringent approach, only selecting studies that measured trauma broadly and categorically and compared two groups. This poses an issue of selective evidence affecting power and preventing any insight into the specific and differential roles of abuse and neglect in the clinical profile, leading in turn to unexpected findings. I hope this commentary contributes to putting the findings into context.
